# Seasonal cercarial emergence patterns of *Opisthorchis viverrini* infecting *Bithynia siamensis goniomphalos* from Vientiane Province, Lao PDR

**DOI:** 10.1186/s13071-014-0551-1

**Published:** 2014-12-02

**Authors:** Nadda Kiatsopit, Paiboon Sithithaworn, Kulthida Kopolrat, Ross H Andrews, Trevor N Petney

**Affiliations:** Department of Parasitology, Faculty of Medicine, Khon Kaen University, Khon Kaen, 40002 Thailand; Liver Fluke and Cholangiocarcinoma Research Center (LFCRC), Faculty of Medicine, Khon Kaen University, Khon Kaen, 40002 Thailand; Cholangiocarcinoma Screening and Care Program (CASCAP), Khon Kaen University, Khon Kaen, 40002 Thailand; Imperial College London, Faculty of Medicine, St Mary’s Campus, South Wharf Street, London, W2 1NY UK; Department of Ecology and Parasitology, University of Karlsruhe, Kornblumen Strasse 13, Karlsruhe, 76131 Germany

**Keywords:** *Opisthorchis viverrini*, *Bithynia siamensis goniomphalos*, Cercarial emergence, Lao PDR

## Abstract

**Background:**

Snail intermediate hosts play a pivotal role in maintaining the life cycles of trematodes, including *Opisthorchis viverrini.* We investigated the emergence patterns of *O. viverrini* cercariae infecting *Bithynia siamensis goniomphalos* at foci in an endemic area in Vientiane Province, Lao PDR.

**Findings:**

Samples of *B. s. goniomphalos* collected during the hot-dry, rainy and cool-dry seasons were examined for *O. viverrini* infection by cercarial shedding. Emergence of cercariae from *O. viverrini*-positive snails was monitored daily from 06:00–18:00 h for seven consecutive days at 2 hourly intervals. Snail infections varied seasonally, being highest in the cool-dry season. Peak cercarial emergence was not consistent in different seasons, occurring between 08.00–10.00 h during the hot-dry season and between 12.00–14.00 h during the rainy and cool-dry seasons. The cercarial output was highest in the hot-dry season. The prevalence of infection and the emergence of cercariae were strongly dependent on snail size.

**Conclusions:**

This study shows that size of snails and environmental factors (such as season) may affect the emergence patterns of cercariae of *O. viverrini* in snails. These results have both fundamental and applied implications for opisthorchiasis epidemiology and control.

## Findings

### Background

The human liver fluke *Opisthorchis viverrini* is an important parasite endemic in Southeast Asian countries along the Mekong River, including Thailand, the Lao People’s Democratic Republic (Lao PDR), southern Vietnam, and Cambodia [1]. An estimated eight million people in Thailand and two million people in Lao PDR are infected with *O. viverrini* [2,3]. Infection caused by this fluke is a major medical problem because it acts as a carcinogen that can cause bile-duct cancer (cholangiocarcinoma, CCA) [4]. *Opisthorchis viverrini* requires freshwater snails and cyprinid fish as first and second intermediate hosts, respectively, to complete its life cycle [5]. The prevalence of infection in the fish hosts is usually very high [6], whereas infection rates in the snail hosts are very low [7,8].

The transmission of many digeneans depends on the ability of the cercariae to invade specific hosts. Within snail hosts, the parasites multiply asexually and produce large numbers of cercariae. The biological characteristics of the cercariae, together with the environmental conditions, play a major role in trematode transmission [9]. *Bithynia* snails are the critical amplifying hosts for *O. viverrini* cercariae and thus are crucial to transmission. The natural prevalence of *O. viverrini* in *Bithynia siamensis goniomphalos* varies between sampling localities and times from 0.22% to 6.93% (mean of 3.03%) in Thailand, and 0.37% to 8.37% (mean of 2.01%) in the Lao PDR [7]. A study of the pattern of emergence of *O. viverrini* cercariae from infected *B. s. goniomphalos* in Thailand under natural conditions found a peak between 08:00–10:00 h with a maximum output of 1,728 cercariae per snail [10].

To date, the cercarial release of *O. viverrini* from *B. s. goniomphalos* from the Lao PDR has not yet been defined. Thus, the aim of this study was to assess the output and seasonal patterns of emergence of *O. viverrini* cercariae from *B. s. goniomphalos* originating from the Nam Ngum River wetland in Vientiane, Lao PDR.

### Methods

#### Sample collection

Samples of *B. s. goniomphalos* (n = 3,650) were collected on three occasions from Vientiane Province, Lao PDR: in April (hot-dry season, average temperature = 28.37°C; average rainfall = 4.75 mm; sunlight = 7.56 h/day; n = 3,267), October (rainy season, average temperature = 27.68°C; average rainfall = 6.44 mm; sunlight = 5.89 h/day; n = 180) and November (cool-dry season; average temperature = 23.4°C; average rainfall = 0.14 mm; sunlight = 8.90 h/day; n = 203). Snails were collected by handpicking and dredging the sediment with a scoop from the same rice field. They were then cleaned, dried and placed into plastic bags and transported to the laboratory at Khon Kaen University where they were identified by standard morphological criteria [11-13]. The shell size (length and width) was measured.

#### Screening of *O. viverrini* infection in *B. s. goniomphalos*

Snail samples were kept under laboratory conditions for one day before screening started. Snails collected from each site were examined by the cercarial shedding method within two days to avoid effect of laboratory maintenance on cercarial emergence [7]. Each snail was placed separately into a small (3 cm in diameter, 2.5 cm in height) plastic container filled with dechlorinated tap water. The containers were covered with a lid studded with pins to prevent the snail from leaving. The snails were exposed to 1,200 lx lamps placed 30 cm above each container for 5 h during the day time at room temperature (25 ± 2°C). *Opisthorchis viverrini* cercariae were identified morphologically using light microscopy. To confirm this morphological identification, PCR analyses were conducted using an *O. viverrini* specific primer and previously published methods [14]. PCR assays were carried out in a final volume of 25 μl consisting of PCR buffer (10 mM Tris–HCl [pH 9], 50 mM KCl, 1.5 mM MgCl_2_), 200 μM each deoxynucleoside triphosphate, 1.5 U Taq DNA polymerase, and 1 μM of each primers (pOV-6). Thermocycling conditions included an initial denaturation at 94°C for 5 min, 30 cycles of 94°C for 30 sec, annealing at 52°C for 30 sec, extension at 72°C for 45 sec, followed by a final extension step at 72°C for 10 min. The amplified PCR products underwent electrophoresis using a 1.5% agarose gel containing 0.1 mg/ml ethidium bromide, and the gel was analyzed using the Gel Documentation System.

#### Emergence of *O. viverrini* cercariae

*Opisthorchis viverrini-*positive snails were maintained for one day in the dark until the experiment started. Infected snails from each sampling period were placed individually into a plastic container filled with 5 ml dechlorinated tap water and exposed to light (1,200 lx lamp placed 30 cm above each container) from 06:00 to 18:00 h at room temperature (25 ± 2°C). Every 2 h the snails were removed to a new container and the cercariae released from each snail over the 2 h interval were counted under a dissecting microscope after staining with 1% Lugol’s iodine solution. At the end of the 12 h period, the snails were returned to the dark for 12 h and fed with ivy gourd leaves, *Coccinia grandis*. They were then exposed to light for 12 h the next day and this pattern continued for seven consecutive days. At the beginning of the new light phase, the containers were checked to determine that no cercariae were shed during the dark phase.

#### Statistical test

Raw data were entered into a Microsoft Excel spreadsheet and descriptive statistics were calculated using SPSS software v. 19.0 (IBM Software Company, USA). Statistical comparisons of cercarial release between different times calculated daily for 7 days and seasons were done using the Two-Related-Sample-Test followed by Wilcoxon Signed Ranks tests. Chi-square tests were used to assess the association of size factors with the prevalence of cercariae. Statistical comparisons of the daily cercarial emergence were done using the Mann–Whitney U test. The results were considered significant when P was <0.05.

### Results

**Figure 1:**
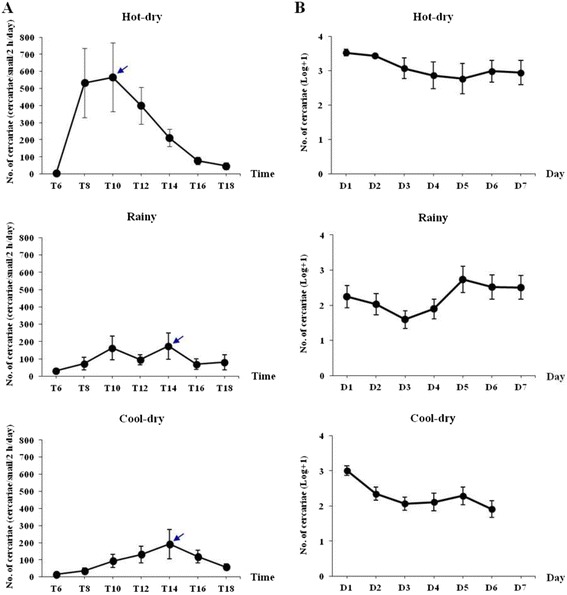
**Cercarial emergence profiles of** ***Opisthorchis viverrini*** **infecting** ***Bithynia siamensis goniomphalos*** **.** Cercarial emergence **(A)** calculated every 2 h from 06:00 until 18:00 h for 7 days (mean ± SE) and **(B)** calculated daily for 7 days (mean ± SE) from snails collected in Lao PDR.

The prevalence of infected snails was 0.21%, 5.00% and 8.37% for the hot-dry, rainy and cool-dry seasons, respectively. A peak in cercarial emergence was observed for the 12.00–14.00 h period in the rainy (mean number of cercariae (±SD) = 174 ± 230 cercariae/snail/2 h/d) and cool-dry (mean number of cercariae = 192 ± 354 cercariae/snail/2 h/d) seasons, whereas in the hot-dry season a peak in emergence was observed for the 08.00–10.00 h period (mean number of cercariae = 567 ± 495 cercariae/snail/2 h/d; Figure 1A). In addition, the snails collected during the hot-dry season showed an increase in cercarial emergence in the morning followed by a decline until evening, whereas emergence in the rainy and cool-dry seasons was lowest in early morning, peaked between 08.00–14.00 h before declining in late afternoon (Figure 1A). The cercarial emergence rates of *O. viverrini* calculated every 2 h from 06:00 until 18:00 h were significantly different between different times and seasons (P <0.05). The mean number of cercariae per snail per day was 263 ± 54.8 cercariae/snail/d for 6 snails in the hot-dry season, 98 ± 75.73 cercariae/snail/d for 9 snails in the rainy season, 91 ± 67.61 cercariae/snail/d for 17 snails in the cool-dry season. The daily cercarial output in both rainy and cool-dry seasons was low until the 3^rd^ day after the start of the experiment, increased during the next 2 days, and decreased during the subsequent 2 days (Figure 1B). In the hot-dry season, the number of cercariae decreased until the 5^th^ day, increased on the 6^th^ and decreased again on the 7^th^. Numbers of emerged cercariae of *O. viverrini* calculated daily for 7 days were significantly different between different days and seasons (P <0.05).

**Figure 2:**
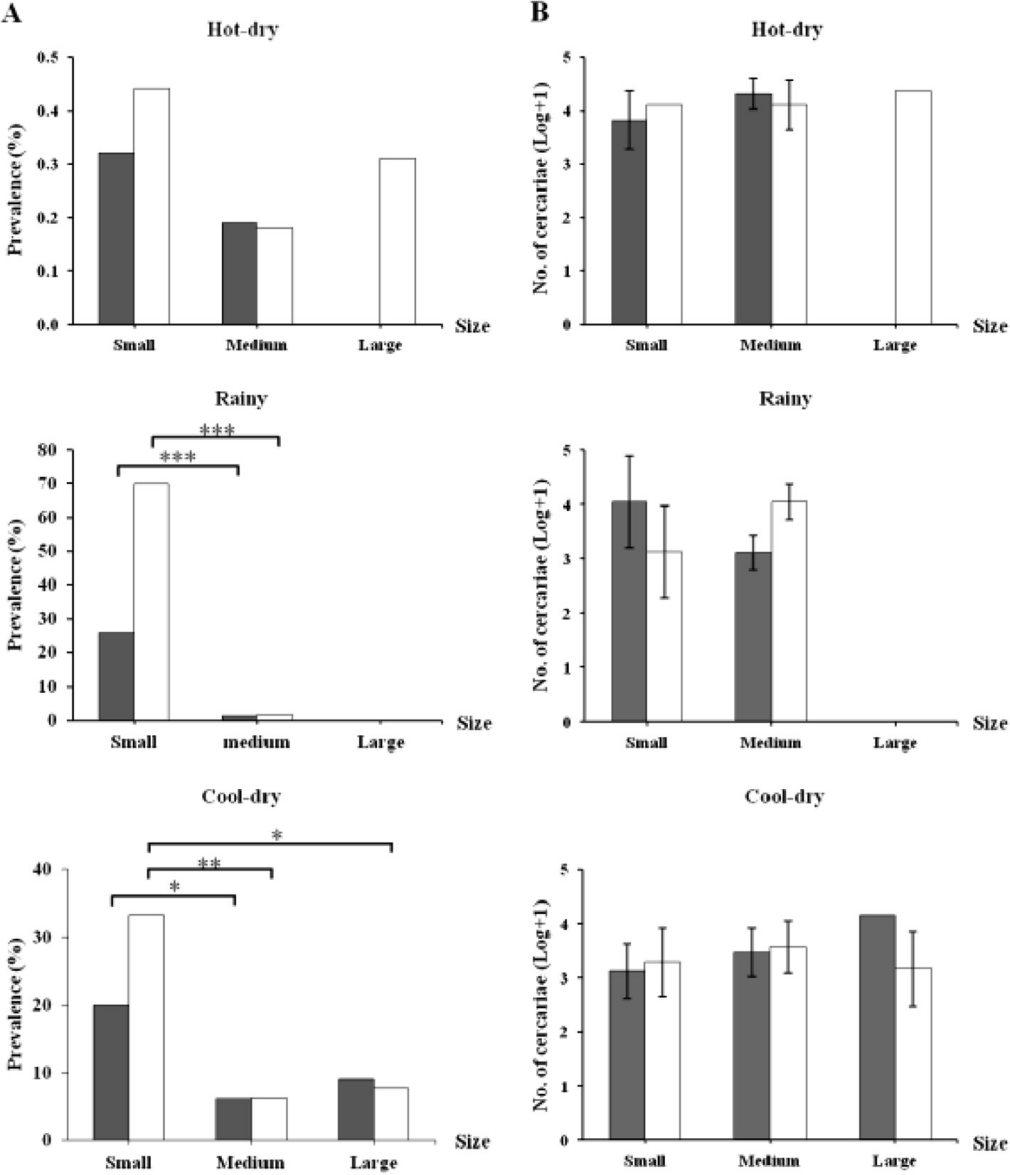
**Relationship between the prevalence (A) and output (B) of** ***Opisthorchis viverrini*** **cercariae and shell-size of** ***Bithynia siamensis goniomphalos*** **.** Mean values (±95% CI) are shown. Black bars represent size-classes based on shell length (small = <8.0 mm, medium-sized = 8.1–10.0 mm, large = >10.0 mm); white bars represent size-classes based on shell width (small = <5.0 mm, medium-sized =5.1–6.0 mm, large = >6.0). *P <0.05, **P <0.01, ***P <0.001. The absence of a bar reflects the lack of infected snails within the corresponding size-class collected.

During the hot-dry season, there were no differences in the prevalence of infection between the small, medium-sized and large snails (Figure 2A)*.* In the rainy season no large snails were collected but small snails had a significantly higher prevalence than the few medium-sized snails with respect to both length (χ^2^_2_ = 21.3, P <0.001) and width (χ^2^_2_ = 36.3, P <0.001). During the cool-dry season, there was a significantly higher cercarial prevalence in small compared to medium-sized snails with respect to both length (χ^2^_2_ = 4.9, P <0.05) and width (χ^2^_2_ = 7.1, P <0.01), and large snails with respect to width (χ^2^_2_ = 4.3, P <0.05). There were no significant differences in the number of cumulative cercarial output with respect to the size of *B. s. goniomphalos* (Figure 2B and Mann–Whitney U tests; all values P >0.05).

### Discussion

*Opisthorchis viverrini* is a typical trematode undergoing asexual reproduction within snail hosts and emergence of the dispersal stages (cercariae) into the water bodies that subsequently infect cyprinid fishes, the second intermediate hosts. The output of cercariae from their snail hosts is a fundamental component of the transmission success in trematodes [15]. This study demonstrates a circadian rhythm in the emergence of the cercariae from their snail hosts, however, the maximum peak of emergence differs according to season. During the hot-dry season, maximum emergence occurs in the morning, whereas it occurs in the afternoon in the rainy and cool-dry seasons. Temporal profiles of cercarial emergence of *O. viverrini* during the hot-dry season showed that maximum output occurred 4 h earlier than during the rainy and cool-dry seasons. In order to avoid possible effects of laboratory maintenance after collection from the field, the snails were processed for cercarial shedding on day 2 after collection and were fed with ivy gourd leaves daily at night time.

*Opisthorchis viverrini* shows a circadian rhythm of cercarial emergence. The adaptive importance of a rhythm in cercarial output is probably related to the facilitation of cercarial transmission to the vertebrate host, making cercarial emergence at different times advantageous [16]. For trematode cercariae, the time when the target host is likely to be present in the same locality is an obvious advantage [17]. This is particularly relevant if one considers the short life-span and the decreasing infectivity of cercariae over time. Such a temporal pattern promotes a high degree of synchrony between parasites and their vertebrate hosts. The cercarial output of *O. viverrini* was higher during daytime, decreased during the evening and no cercariae emerged at night. Parasites may use a range of strategies to maximize transmission efficiency in an environment where the presence of the desired host is uncertain or temporally aggregated [18]. Alternatively, the observed higher output of cercariae during daytime, when most of the fish hosts are active [19,20] and more likely to be exposed to cercariae, may increase parasite transmission efficiency.

The prevalence of *O. viverrini* in snails from the Lao PDR was highest in the cool-dry season. Similarly, our preliminary investigations indicate that the prevalence of *O. viverrini* in *B. s. goniomphalos* from Sakon Nakhon Province, Thailand tends to be high in the cool-dry months (Kopolrat *et al*., unpublished data). Whether this is in fact the case requires more intensive investigation. The faecal bacterial contamination in natural water reservoirs is an important indicator of seasonal transmission of *O. viverrini* eggs to snail intermediate hosts occurring in the rainy season [21]. After acquisition of infection, the snails require about two months for larval development before the emergence of free-swimming cercariae [22,23]. In addition, higher prevalence rates of *O. viverrini* infection in the snails in the cool-dry season corresponded to high burdens of *O. viverrini* in infected cyprinid fish in the late rainy and cool-dry seasons [24].

In our study, the cercarial output of *O. viverrini* was very high in the hot-dry season. Seasonal factors can affect the number of cercariae produced. For example, seasonal differences in the numbers of *Schistosoma mansoni* cercariae per *Biomphalaria alexandrina* in the Nile Delta were highest from June to September (summer) and declined to zero in February and March (winter and spring) [25]. Similarly, for both *Rhipidocotyle fennica* in clams and *Diplostomun spathaceum* in *Lymnaea stagnalis* in northern temperate climates the number of emerged cercariae was highest in late summer [26,27]. In a preliminary study by Kopolrat and others (unpublished data), *O. viverrini* cercarial output from *B. s. goniomphalos* collected from Sakon Nakhon Province at different times over a four year periods was highest in the hot-dry season (equivalent to summer) as is the case for the *O. viverrini* population from the Lao PDR studied here.

Cercarial output may be enhanced at higher temperatures and light as a simple consequence of increased host metabolic activity resulting in the greater energy resources available to the parasite [15,28]. Schmidt and Fried [29] found that the only significant factors in the emergence of *Echinostoma trivolvis* cercariae from naturally infected *Helisoma trivolvis* snails that were maintained under different laboratory conditions were temperature related. In their review, Galaktionov and Dobrovolskij [30] indicate that light and temperature are the predominant controlling factors for the emergence of cercariae. Kaewkes and others [31] found that a light intensity of 1,000 lx was the most important stimulus for *O. viverrini* cercariae emerging from naturally infected *B. s. goniomphalos* snails in the laboratory.

The rate of development of the trematode in its snail host is dependent on the access to ample supplies of food [32]. The feeding of snails accelerates parasite maturity so that at a given time many more mature cercariae are formed in well-fed, larger snails than smaller ones [33,34]. This is in contrast to the finding of Schmidt and Fried [29] who studied cercarial output of *E. trivolvis* from naturally infected *H. trivolvis* snails maintained under different laboratory conditions and found that food had no affect on cercarial emergence. However, other factors such as the size of the snails can also affect cercarial output [7].

Our initial analyses indicate that there is a possible relationship between the prevalence of *O. viverrini* cercariae and shell size, with the prevalence of *O. viverrini* in small snails being significantly higher than in medium-sized and large snails. This could be a result of higher susceptibility to infection of smaller snails than of larger snails and also of parasite-induced snail mortality [35,36], as large snails may have acquired multiple infections or may have been infected for a longer period. The survival time of large snails (*Lymnaea peregra*) infected with *Echinoparyphium recurvatum* was found to be shorter than for small infected snails [37].

### Conclusions

The size of the snails and environmental factors influencing cercarial emergence patterns were reported for the first time. The prevalence of *O. viverrini* in field-collected snail intermediate hosts, originating from Vientiane, Lao PDR was highest in the cool-dry season and commonly found in small snails. Cercarial output was highest in the hot-dry season. These results provide important fundamental and applied implications for control of opisthorchiasis in the Lao PDR. Whether these observed data are associated with different strains of *O. viverrini* s.l. in the Lao PDR and Thailand is not known. This hypothesis requires further investigation to compare the cercariae pattern of *O. viverrini* s.l. from Lao PDR and Thailand.

## References

[1] Andrews RH, Sithithaworn P, Petney TN (2008). *Opisthorchis viverrini*: an underestimated parasite in world health. Trends Parasitol.

[2] Sithithaworn P, Andrews RH, Petney TN, Saijuntha W, Laoprom N (2012). The systematics and population genetics of *Opisthorchis viverrini* sensu lato: implications in parasite epidemiology and bile duct cancer. Parasitol Int.

[3] Phongluxa K, Xayaseng V, Vonghachack Y, Akkhavong K, van Eeuwijk P, Odermatt P (2013). Helminth infection in southern Laos: high prevalence and low awareness. Parasit Vectors.

[4] Sithithaworn P, Yongvanit P, Duenngai K, Kiatsopit N, Pairojkul C (2014). Roles of liver fluke infection as risk factor for cholangiocarcinoma. J Hepatobiliary Pancreat Sci.

[5] Sithithaworn P, Andrews RH, Nguyen VD, Wongsaroj T, Sinuon M, Odermatt P, Nawa Y, Liang S, Brindley PJ, Sripa B (2012). The current status of opisthorchiasis and clonorchiasis in the Mekong Basin. Parasitol Int.

[6] Saijuntha W, Sithithaworn P, Kaitsopit N, Andrews RH, Petney TN (2014). Liver flukes: clonorchis and opisthorchis. Adv Exp Med Biol.

[7] Kiatsopit N, Sithithaworn P, Saijuntha W, Boonmars T, Tesana S, Sithithaworn J, Petney TN, Andrews RH (2012). Exceptionally high prevalence of infection of *Bithynia siamensis goniomphalo*s with *Opisthorchis viverrini* cercariae in different wetlands in Thailand and Lao PDR. Am J Trop Med Hyg.

[8] Petney T, Sithithaworn P, Andrews R, Kiatsopit N, Tesana S, Grundy-Warr C, Ziegler A (2012). The ecology of the *Bithynia* first intermediate hosts of *Opisthorchis viverrini*. Parasitol Int.

[9] Haas W (1994). Physiological analyses of host-finding behaviour in trematode cercariae: adaptations for transmission success. Parasitology.

[10] Phongsasakulchoti P, Sri-aroon P, Kerdpuech Y (2005). Emergence of *Opisthorchis viverrini* cercariae from naturally infected *Bithynia* (*Digoniostoma*) *siamensis goniomphalos*. Southeast Asian J Trop Med Public Health.

[11] Brandt RAM (1974). The non-marine aquatic Mollusca of Thailand. Archiv für Molluskenkunde.

[12] Chitramvong YP (1992). The Bithyniidae (Gastropoda: Prosobranchia) of Thailand:comparative external morphology. Malacol Rev.

[13] Upatham ES, Sornmani S, Kitikoon V, Lohachit C, Bruch JB (1983). Identification key for fresh-brackish water snails of Thailand. Malacol Rev.

[14] Wongratanacheewin S, Pumidonming W, Sermswan RW, Maleewong W (2001). Development of a PCR-based method for the detection of *Opisthorchis viverrini* in experimentally infected hamsters. Parasitology.

[15] Poulin R (2006). Global warming and temperature-mediated increases in cercarial emergence in trematode parasites. Parasitology.

[16] Combes C, Bartoli P, Théron A, Lewis EE, Campbell JF, Sukhdeo MVK (2002). Trematode Transmission Strategies. The Behavioural Ecology of Parasites.

[17] Théron A, Combes C (1995). Asynchrony of infection timing, habitat preference, and sympatric speciation of *Schistosome* parasites. Evolution.

[18] Fenton A, Hudson PJ (2002). Optimal infection strategies: should macroparasites hedge their bets?. Oikos.

[19] Helfman G, Pitcher TJ (1986). Fish Behaviour by Day, Night and Twilight. The Behaviour of Teleost Fishes.

[20] Wieser W, Winfield IJ, Nelson JS (1991). Physiological Energetics and Ecophysiology. Cyprinid Fishes. Volume 3.

[21] Kaewkes W, Kaewkes S, Tesana S, Laha T, Sripa B (2012). Fecal bacterial contamination in natural water reservoirs as an indicator of seasonal infection by *Opisthorchis viverrini* in snail intermediate hosts. Parasitol Int.

[22] Harinasuta C, Harinasuta T (1984). *Opisthorchis viverrini*: life cycle, intermediate hosts, transmission to man and geographical distribution in Thailand. Arzneimittelforschung.

[23] Upatham ES, Viyanant V (2003). *Opisthorchis viverrini* and opisthorchiasis: a historical review and future perspective. Acta Trop.

[24] Sithithaworn P, Pipitgool V, Srisawangwong T, Elkins DB, Haswell-Elkins MR (1997). Seasonal variation of *Opisthorchis viverrini* infection in cyprinoid fish in north-east Thailand: implications for parasite control and food safety. Bull World Health Organ.

[25] Chu KY, Dawood IK (1970). Cercarial transmission seasons of *Schistosoma mansoni* in the Nile Delta area. Bull World Health Organ.

[26] Taskinen J (1998). Cercarial production of the trematode *Rhipidocotyle fennica* in clams kept in the field. J Parasitol.

[27] Karvonen A, Savolainen M, Seppala O, Valtonen ET (2006). Dynamics of *Diplostomum spathaceum* infection in snail hosts at a fish farm. Parasitol Res.

[28] Mas-Coma S, Valero MA, Bargues MD (2009). Climate change effects on trematodiases, with emphasis on zoonotic fascioliasis and schistosomiasis. Vet Parasitol.

[29] Schmidt KA, Fried B (1996). Emergence of cercariae of *Echinostoma trivolvis* from *Helisoma trivolvis* under different conditions. J Parasitol.

[30] Galaktionov KV, Dobrovolskij AA (2003). The Biology and Evolution of Trematodes.

[31] Kaewkes S, Kaewkes W, Boonmars T, Sripa B (2012). Effect of light intensity on *Opisthorchis viverrini* cercarial shedding levels from *Bithynia* snails–a preliminary study. Parasitol Int.

[32] Kendall SB (1949). Nutritional factors affecting the rate of development of *Fasciola hepatica* in *Limnaea truncatum*. J Helminthol.

[33] Belfaiza M, Rondelaud D, Moncef M, Dreyfuss G (2004). *Fasciola hepatica*: the effect of food quality on the development of redial generations in *Galba truncatula* infected with allopatric miracidia. Parasitol Res.

[34] Seppala O, Liljeroos K, Karvonen A, Jokela J (2008). Host condition as a constraint for parasite reproduction. Oikos.

[35] Fredensborg BL, Mouritsen KN, Poulin R (2005). Impact of trematodes on host survival and population density in the intertidal gastropod *Zeacumantus subcarinatus*. Mar Ecol Prog Ser.

[36] Sorensen RE, Minchella DJ (1998). Parasite influences on host life history: *Echinostoma revolutum* parasitism of *Lymnea elodes* snails. Oecologia.

[37] Morley NJ, Adam ME, Lewis JW (2010). The effects of host size and temperature on the emergence of *Echinoparyphium recurvatum* cercariae from *Lymnaea peregra* under natural light conditions. J Helminthol.

